# A Review on Computer Aided Diagnosis of Acute Brain Stroke

**DOI:** 10.3390/s21248507

**Published:** 2021-12-20

**Authors:** Mahesh Anil Inamdar, Udupi Raghavendra, Anjan Gudigar, Yashas Chakole, Ajay Hegde, Girish R. Menon, Prabal Barua, Elizabeth Emma Palmer, Kang Hao Cheong, Wai Yee Chan, Edward J. Ciaccio, U. Rajendra Acharya

**Affiliations:** 1 Department of Mechatronics, Manipal Institute of Technology, Manipal Academy of Higher Education, Manipal 576104, India; mahesh.inamdar@manipal.edu; 2 Department of Instrumentation and Control Engineering, Manipal Institute of Technology, Manipal Academy of Higher Education, Manipal 576104, India; anjan.gudigar@manipal.edu (A.G.); yashaschakole1999@gmail.com (Y.C.); 3 Department of Neurosurgery, Kasturba Medical College, Manipal Academy of Higher Education, Manipal 576104, India; dr.ajayhegde@gmail.com (A.H.); girish.menon@manipal.edu (G.R.M.); 4 School of Management & Enterprise, University of Southern Queensland, Toowoomba, QLD 4350, Australia; prabal.barua@usq.edu.au; 5 Faculty of Engineering and Information Technology, University of Technology, Sydney, NSW 2007, Australia; 6 Cogninet Brain Team, Cogninet Australia, Sydney, NSW 2010, Australia; 7 School of Women’s and Children’s Health, University of New South Wales, Sydney, NSW 2052, Australia; elizabeth.palmer@health.nsw.gov.au; 8 Science, Mathematics and Technology Cluster, Singapore University of Technology and Design, Singapore 487372, Singapore; kanghao_cheong@sutd.edu.sg; 9 Department of Biomedical Imaging, Research Imaging Centre, University of Malaya, Kuala Lumpur 59100, Malaysia; waiyeec@ummc.edu.my; 10 Department of Medicine, Columbia University, New York, NY 10032, USA; ciaccio@columbia.edu; 11 Department of Biomedical Engineering, Faculty of Engineering, University of Malaya, Kuala Lumpur 50603, Malaysia; aru@np.edu.sg; 12 School of Engineering, Ngee Ann Polytechnic, Singapore 599489, Singapore; 13 Department of Biomedical Engineering, School of Science and Technology, SUSS University, Singapore 599491, Singapore; 14 Department of Biomedical Informatics and Medical Engineering, Asia University, Taichung 41354, Taiwan

**Keywords:** Ischemic brain stroke, machine learning, deep learning, CAD

## Abstract

Amongst the most common causes of death globally, stroke is one of top three affecting over 100 million people worldwide annually. There are two classes of stroke, namely ischemic stroke (due to impairment of blood supply, accounting for ~70% of all strokes) and hemorrhagic stroke (due to bleeding), both of which can result, if untreated, in permanently damaged brain tissue. The discovery that the affected brain tissue (i.e., ‘ischemic penumbra’) can be salvaged from permanent damage and the bourgeoning growth in computer aided diagnosis has led to major advances in stroke management. Abiding to the Preferred Reporting Items for Systematic Review and Meta–Analyses (PRISMA) guidelines, we have surveyed a total of 177 research papers published between 2010 and 2021 to highlight the current status and challenges faced by computer aided diagnosis (CAD), machine learning (ML) and deep learning (DL) based techniques for CT and MRI as prime modalities for stroke detection and lesion region segmentation. This work concludes by showcasing the current requirement of this domain, the preferred modality, and prospective research areas.

## 1. Introduction

In the recent past, stroke has become the foremost cause of mortality and health–disability worldwide, causing over 6.6 million deaths annually [1], and with up to 50% of survivors being chronically disabled [2]. Economic impacts post–treatment and for post–stroke care are substantial [1,2]. Risk factors for stroke are both non–modifiable/inherent and modifiable. Former risk factors include age, gender and genetic factors: with stroke incidence being higher in older individuals and men [3]. Hypertension, smoking, high alcohol consumption, waist–to–hip ratio, and diet are amongst the modifiable risk factor for stroke, particularly ischemic stroke [4]. 

**Figure 1:**
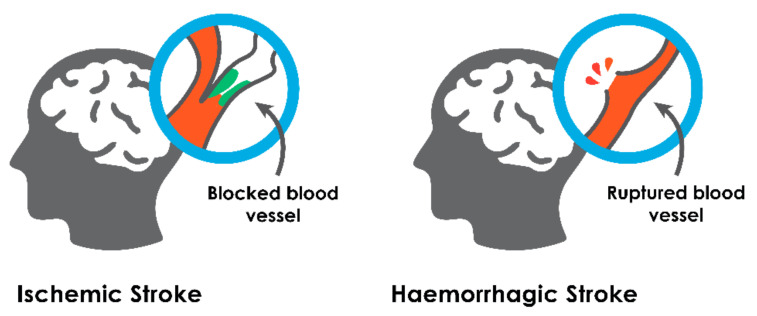
Ischemic and hemorrhagic brain stroke.

Brain strokes are of two types: ischemic (due to intracerebral vessel occlusion) and hemorrhagic (due to intracerebral bleeding), as depicted in Figure 1.

**Figure 2:**
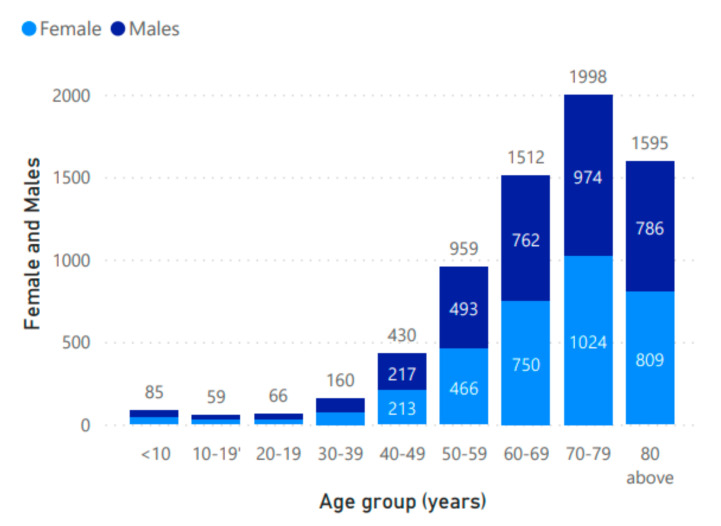
Age–specific incidence rate of strokes by gender in India, 2019.

Ischemic strokes are globally more common, accounting for at least 70% of all strokes. It is most often caused by thrombosis (clots) secondary to large artery atherosclerosis, or emboli from the heart in cardiac disease, such as atrial fibrillation [5]. Other causes of ischemic stroke are small vessel disease, arterial dissections (tears), vasculitis, hypotensive vasoconstriction [5], and hematological disorders (for example, sickle cell anemia, which is a leading cause of pediatric stroke in Africa) [6]. Epidemiological patterns vary between countries. The reductions in incidence, mortality, and disability–adjusted life years in ischemic strokes in high–income countries, largely attributed to improved lifestyles and rapid detection and treatments, has not yet been seen in low and middle–income countries [7]. This widening health inequity is a major challenge, which needs to be urgently tackled, and more emphasis must to be given to democratize cost effective AI solutions for diagnosis and stratifications. In India, the largest contributors to total neurological disorder were strokes (37.9%), causing 7.4% of total deaths [8]. Stratification by gender for the Indian population is shown in Figure 2.

Although hemorrhagic strokes account for 10–15% of total stroke incidence, they are associated with very high mortality and morbidity, which has not improved globally over the last 20 years. Mortality is >50% with half of the deaths occurring in the first two days. Only 20% of patients manage to gain independent activity daily living (ADL) six months after a hemorrhagic stroke. Uncontrolled hypertension and vascular malfunction are the leading causes of hemorrhagic stokes [9,10].

Since the 1970s, it has been recognized that following an acute stroke there are hypo–perfused brain regions, the ‘penumbral regions’, which are supplied by alternative blood flow pathways to the one which is occluded. These penumbral regions can potentially be rescued from permanent cell death if identified in time for reperfusion therapy (restoration of normal blood supply), as well as treatments for the underlying cause of an individual’s stroke [11]. For ischemic stroke due to thromboembolism (the most common cause of strokes) this would include intravenous thrombolysis medication therapy. The time window for a successful and most effective treatment is very small [12]. For example, best outcomes for the thrombolytic medication alteplase are achieved when this is started within 3 h [13]. This ultra–rapid, tailored management approach, ideally within dedicated stroke units, is a key recommendation of the World Stroke Organization’s global guidelines, and essential in reducing mortality and morbidity [14]. 

However, intravenous thrombolysis is contraindicated if there is presence of hemorrhage in the infarcted area. Therefore, stroke guidelines recommend that all patients who are candidates for reperfusion therapies should undergo immediate neuroimaging, ideally with MRI, and all other suspected stroke patients should have an urgent brain computed tomogram (CT) or magnetic resonance imaging (MRI), preferably within 60 min [15]. Moreover, if reperfusion therapy has been escalated, repeat neuroimaging, approximately 24 h after therapy administration, is recommended to identify early complication such as hemorrhagic transformation, and to reassess the infarcted core. This is crucial to decide the next appropriate treatments (for example, anti–thrombotic therapies and deep vein thrombosis prophylaxis) [16]. 

**Figure 3:**
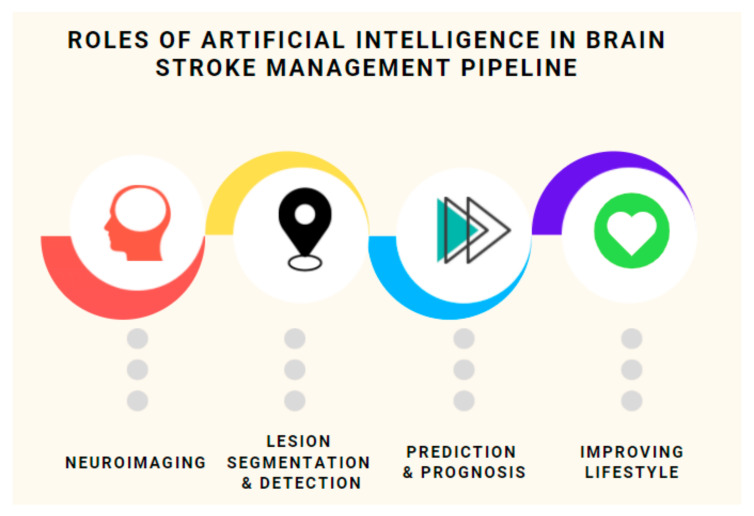
Schematic to showcase applications of AI in stroke management.

Thus, accurate interpretation of neuroimaging to guide most appropriate treatment decisions (‘image–based treatment guidance’) is a key part of modern optimal stroke management. This can be very challenging, and dependent on highly skilled radiologists working under intense time pressures, who are able to carefully analyze a very large amount of radiological data to accurately identify normal and infarcted regions (‘lesion segmentation’), as well as underlying pathophysiological mechanisms such as arterial occlusions. The optimal approach to neuroimaging analysis has been intensively studied, and the possibility of incorporation of artificial intelligence, machine learning (ML), and deep learning (DL), has been increasingly explored [17]. Figure 3 shows the current role AI plays in the brain stroke process.

Both CT and MRI are used in stroke management globally [18]. A more time–saving imaging modality is CT, and this is usually the first line of imaging. MRIs, on the other hand, employ electromagnetic waves to produce superior image resolution of the brain. An MRI with diffusion weighted sequence (DWI) is able to identify hyper–acute stroke events as early as within minutes of onset [19]. Thus, although CT scanners are more accessible globally, less expensive and quicker than MRIs, MRIs are more sensitive to detect acute ischemic events, and thus recommended (especially when there is a potential for reperfusion therapy) [20,21]. However, recent developments in CT technology, such as perfusion CT (CTP) to identify ischemic penumbra combined with CT angiography (CTA) to identify occluded vessels, represent promising alternatives to allow fast response and associated assessment of stroke patients in cases where MRI machines are either too expensive, inaccessible, or contraindicated [22]. 

The review articles in the literature have extensively investigated and discussed the recent advancements in neuroimaging, various techniques employed for detection and lesion segmentation (lesion stage wise by Yue), and the challenges involved in these techniques [23,24,25,26,27]. Although these studies have contributed tremendously, there is still scope for a deeper and more exhaustive study particularly based on modalities and their suitability to the current conditions. Moreover, we have identified that there was a need for a single comprehensive work amalgamating details of the recent development in all areas of this domain.

### 1.1. Review Objective

The main objective of this paper, besides discussing the most recent developments in diagnostic techniques, is to provide the reader with:

- A comprehensive overview of various modalities involved in neuroimaging, their characteristics, and requirement. We compare the most prominent ones and make remarks on their suitability, accessibility and viability. This will be useful in prioritizing future research avenues;
- An all–inclusive overview of a host of recent techniques (with special focus on prognosis) for stroke classification, detection and lesion segmentation categorized on the basis of modality used, techniques employed, datasets used (with benchmarks) (see Table 1) and the challenges faced;
- The areas of plausible future research.

### 1.2. Article Search

A systematic literature review was performed following PRISMA guidelines. A comprehensive database search was conducted to identify peer–reviewed articles published between 2010 and 2021 including the following terms ‘Ischemic Stroke’, ’Hemorrhagic Stroke’, ‘Lesion Segmentation’, ‘Prognosis of Brain Strokes’, ‘Lesion Detection and Segmentation’, ‘Penumbra Core’ and ‘Neuroimaging’. Search engines used were: Science Direct, IEEE Xplore, Spice, Springer and Wiley.

### 1.3. Selection of Articles

**Figure 4:**
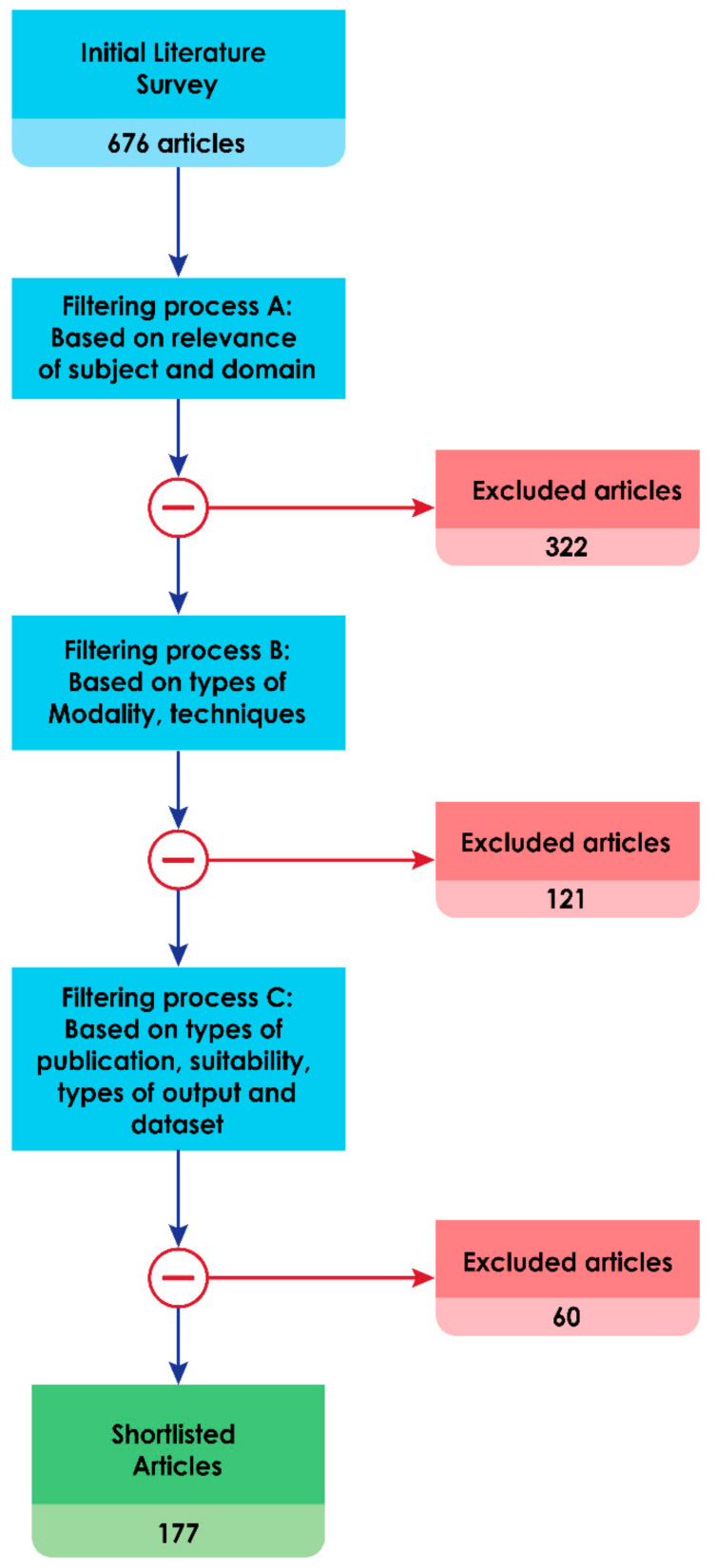
Articles selection process based on the PRISMA guidelines.

Studies published between 2010 and 2021 which strictly adhere to the subject area and a few earlier survey methods, case definitions and concept studies were considered. The entire process was carried out with three level filtering. 676 articles were initially collected and 322 publications were filtered out as they were not relevant to the subject and domain. A further 121 were filtered out due to the type of methods employed. 177 were finally shortlisted for analysis based on relevance, type of publication, modality and implementation details of technical aspects. Figure 4 shows articles selection process. To the best of our knowledge, we have gathered all publications between 2010 and 2021 covering this subject.

This systematic review has been written following the PRISMA guidelines. The inclusion and exclusion criteria are mentioned in Table 2.

### 1.4. Analysis of Articles

177 papers which met the inclusion and exclusion criteria were analyzed and stratified based on imaging modality, techniques applied (ML/DL), and the types of problem addressed.

### 1.5. Paper Structure

**Figure 5:**
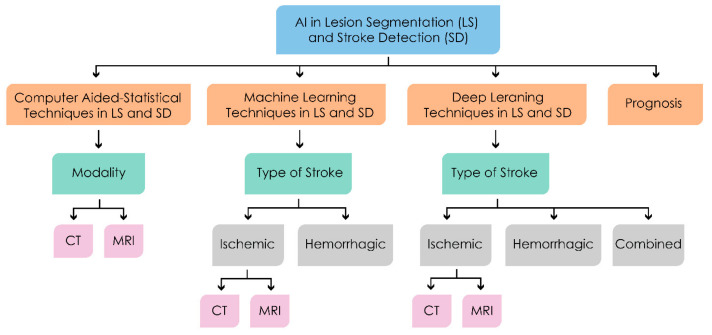
Structure of the review process.

Here, we mention the structure of the paper. In Section 2, a brief overview is provided of the basics of imaging in the brain stroke domain. It begins by describing the considered modalities with their description and working principles. Section 4 contains the prospects of deep learning and a comprehensive review of various techniques/architectures for stroke (ischemic/hemorrhagic) detection and lesion region segmentation and prognosis. In Section 5 we discuss learning, research gaps, and future scope. Finally, Section 6 contains the compendium of the research work. Figure 5 shows the structure of this paper.

## 2. Brief Perspective on Brain Stroke Imaging

Neurological abnormalities are captured through CT and MRI. CT perfusion imaging has been used to assess the degree of the ischemic penumbra, infarcted core and to aid treatment decision. CT in the acute setting is mainly to identify contraindication to thrombolysis and to exclude stroke mimics. In this section we present a brief overview of stroke imaging techniques and the modalities employed for diagnosis and treatment. Table 3 and Figure 5 provide detailed description of the considered modalities.

### 2.1. Ischemic Stroke

Reduced cerebral blood flow (CBF) due to the occlusion of blood vessels lead to ischemic stroke. Although ischemia tolerance differs between tissue types, these types of strokes can be fatal when there is large vessel occlusion [28]. The deprived tissue begins losing essential nutrients and oxygen and excretes toxins which accumulate and impact normal function. Failure of recanalization of blood vessels ultimately can lead to tissue infarction (death) [28]. Features such as hyper–dense middle cerebral artery (MCA), cerebral swelling caused by sulcal or ventricular effacement, and focal parenchymal hypo–attenuation are most relevant to stroke assessment [29]. 

**Figure 6:**
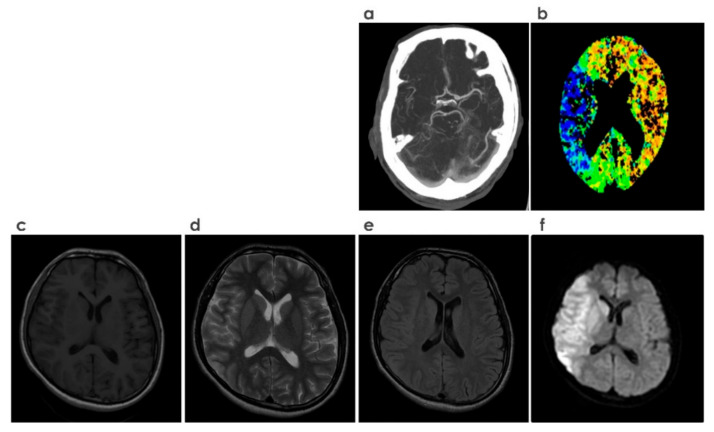
Various neuroimaging modalities. (**a**) CT Angiography, (**b**) CT Perfusion, (**c**) T1–weighted imaging, (**d**) T2–weighted imaging, (**e**) FLAIR (fluid attenuated inversion recovery), (**f**) DWI (diffusion weighted imaging).

Ischemic core volume on baseline non–contrast CT (NCCT), CT perfusion (CTP), or diffusion–weighted magnetic resonance imaging (DWI) (Figure 6 shows the comparison of these modalities) is now widely used to drive key therapeutic decisions both in the early and late (beyond 6 h after last known time well) time windows [30]. Besides accessibility, speed, and patient tolerance, NCCT, when viewed in appropriate window width and window length, can detect early hyper–acute ischemic alterations which helps to predict both final outcome and the risk of secondary hemorrhagic changes [31]. DWI has been shown to contribute significantly to the early detection of acute ischemic infarction, which can be seen as a hyper–intense signal, due to the drop in diffusivity [32].

### 2.2. Hemorrhagic Stroke

Spontaneous extravasation of blood due to rupture of a vessel causes hemorrhagic stroke. The CT appearance of hemorrhage is proportional to the density of hemoglobin protein (relative to plasma concentrations) within the hematoma. Immediately following a vessel rupture, attenuation of CT is given in terms of Hounsfield units, which is a linear, quantitative measurement of radio density [33]. Contrast–enhanced CT angiogram (CTA) can identify patients at high risk of hemorrhage enlargement (HE) by revealing a ‘spot sign’, which indicates an active bleeding point within the hematoma. MRI, on the other hand, can detect previously resolved bleed and clinically silent cerebral microbleeds that are not detectable on CT. This is due to the detection of hemosiderin which is an end result of blood clot resolution [34]. Detection of hemosiderin provides information on previous history of hemorrhages that may have gone undetected [35]. Table 4 presents the radiological features shown by the considered modalities in different classes of stroke.

## 3. Machine Intelligence in Lesion Segmentation and Stroke Detection

Lesion segmentation and identification of brain abnormality has long been a subject of research, and many resulting developments have been made. Computer aided techniques with statistical analysis have improved the process and model accuracy. However, as many of these tools rely on human intervention or for crafting specialized features, these methods are computationally expensive and suffer from a lack of generalizability. In contrast, machine learning algorithms can learn from hidden data and offer great flexibility. However, these too have the problem of addressing handcrafted features and being specific to the available dataset. Hence, it is prudent to develop a technique with many parameters to learn and acquire the important features, thereby sparing manual work. Introduction of such systems in medical practice, if accurate and sensitive, may be cost effective, freeing clinicians to focus on other areas of patient management. In regions where skilled radiologists are limited, an automated technique can improve accessibility and equity in high quality medical care. Most importantly, they have the ability to improve the early detection of stroke and facilitate improved outcomes guided by accurate neuroimaging [36].

These deep learning networks are known as global function approximators, making them ideal tools for the case with non–uniform relationships between parameters. As these have learnable parameters in multitude, they are highly efficient in capturing minute and salient information [37]. However, there are some difficulties, the first being that they are ‘data hungry’, and sufficient data may, in some cases, be difficult to provide, leading to the need for data augmentation. Secondly most networks are massive in terms of layers and hence the changes in derivatives might not effectively initiate earlier neurons. Thirdly, as the computations depend on decision logic, higher–level computational machinery is required, such as the use of graphics processing units (GPUs) or tensor processing units (TPUs). Due to rapid growth in healthcare and computational infrastructure, deep learning in many ways has stood the test of time to emerge as an efficient tool for such applications. 

The following sections provide an overview on the current techniques with CT and MRI as primary modalities for ischemic and hemorrhage stroke detection.

### 3.1. Computer Aided—Statistical Techniques

Schemes with detection technique aided by computer processing (CAD) can help identify patterns or abnormality that might be missed in preliminary clinical diagnosis, and with the automatic feature extraction may improve disease detection. These can be broadly grouped into two types: (a) region of interest (ROI) detection followed by stroke prediction, and (b) segmentation. In the following section we discuss relevant papers in the same order.

#### 3.1.1. CT Based Methods

Techniques from the past decade have evolved tremendously from region identification, feature extraction through image enhancement through computer to identify stroke, and early detection which is critical to guide most appropriate therapies and improve health care outcomes. Tang et al., proposed a way to isolate the region of interest of geometric shapes to analyze CT scans for prompt discovery of ischemic stroke [38]. The algorithm contains a series of filters using radii of pixel to obtain the region of interest and produce a binary mask. The technique performs the identification for detecting brain midline using statistical analysis. Sajjadi et al., proposed a filter bank algorithm (adaptive partial median filer), called the àtrous algorithm to clear the noise and enhance the image for detecting early signs of ischemic stroke [39]. 

To deal with case of misses by experts, due to the low sensitivity of NCCT in detecting cerebral infarctions, Nowinski et al., proposed a quick, less intensive and automatic method to detect, isolate and assess ischemic infarct from a single NCCT scan [40]. Filho et al., proposed a method based on extracting radiological density patterns of the brain to detect and categorize the occurrence of stroke. Five classifiers were applied and compared for ischemic stroke detection in CT images [41]. Flottman et al., experimented with threshold–free prediction of brain infarct from CTP imaging in case of core to penumbra lesion mismatch [42]. Sakai et al., compared Bayesian versus singular value deconvolution for estimation of ischemic core volume as a discriminant. He used a CTP–CBF threshold <30% of a normal brain [43]. As early identification of stroke can be tremendously advantageous, Lo et al., developed a feature set extracted and enhanced by the Ranklet transform to feed the ML classifier for the early detection of hyper–acute ischemic stroke [44]. Shervin K et al., presented a study to determine the finest CTP parameters and associated threshold to clearly discriminate between benign and at–risk penumbra region without reperfusion [45]. Kheradmand et al., conducted a study which showed that in cases of operfusion CT, Time to peak when compared with mean transit time is a more accurate parameter to identify the “at–risk” tissue [46]. Kawiorski M et al., conducted a study which emphasizes the utility of perfusion CT to identify the potentially salvageable tissues by corroborating the presence of correlation between the clinical and radiological aspects [47].

Bhaduria et al., presented a unique region growing based segmentation technique wherein fuzzy c mean facilitates identification of active contour and thereafter propagation through region–based method for intracranial hemorrhage (ICH) detection [48]. Haan et al., proposed the clusterize algorithm as a semi–automated lesion segmentation approach to speed up the demarcation process without reducing precision [49]. Yahiaoui et al., enhanced brain CT imagery using the Laplacian pyramid (LP) and then a Fuzzy C mean clustering algorithm for segmentation of ischemic stroke [50]. In grayscale threshold–based techniques, Reboucas et al., proposed a new and more stable level set approach for stroke segmentation in CT brain imagery [51]. Kumar et al., proposed an entropy based unsupervised segmentation techniques for brain ICH [52]. Vasconcelos et al., proposed a faster method for extracting featuring using adaptive brain tissue density analysis coupled with federated learning to aid in stroke detection and classification [53].

#### 3.1.2. MRI Based Methods

**Figure 7:**
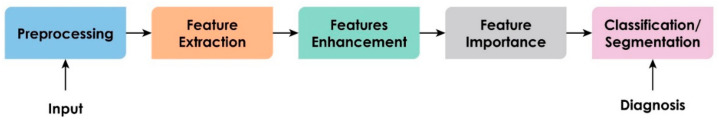
General block diagram of a typical ML–based CAD system.

A standard MRI for acute stroke protocol consists of multiple sequences, from basic T1–weighted, T2–weighted, fluid attenuated inversion recovery (FLAIR), diffusion weighted imaging (DWI), susceptibility–weighted imaging (SWI) and MR angiography (MRA) (doi:10.1148/rg.325115760). This multispectral application is time consuming. In this regard, Nabizadeh et al., proposed an intensity–based segmentation technique optimized by gravitational algorithm for automatic stroke detection and segmentation using single–spectral MRI [54]. Ghosh et al., performed comparative analysis of three techniques for segmentation, namely modified watershed segmentation (MWS), symmetry integrated region growing (SIRG), and hierarchical region splitting (HRS) for the detection of hypoxic ischemic injuries [55]. In the case of segmentation, a lack of a sharp boundary delineation hinders and delays the identification process. Cauley et al., tested a hypothesis and proved that image intensity inhomogeneity provides a sign for identifying the subtle hypo–density regionals which, in turn, is characteristic of ischemic infarct [56]. Ledig C et al., proposed a probabilistic framework for automatic segmentation of MRI using ‘‘multi–atlas label propagation” [57]. Farsani et al., proposed a fully automated lesion segmentation method, which works on diffusion restriction characteristics of the acute stroke images [58]. Moeskops et al., proposed a voxel based automatic segmentation into several tissue classes using CNN with different patch sizes and kernel sizes to acquire multi–scale information about each voxel [59]. Oula et al., presented a simultaneous processing approach which combines brain segmenting techniques with a novel spatial lesion model for identifying distinct brain structures using a restricted Boltzmann machine [60]. Si et al., proposed a wavelet transform based supervised segmentation technique optimized by Grammatical Bee Colony algorithm [61]. Tom et al., presented a probabilistic technique for calculating intensities of both normal and at risk (pathological) tissue without the need of a training set [62]. Ji et al., performed accurate segmentation of brain tissue from the MR image based Gaussian mixture model (GMM) [63]. Kamnitsas et al., proposed an architecture which addresses the challenging task of brain lesion segmentation, making it more efficient and adaptive to the class imbalance problem [64]. Figure 7 presents a generalized pictorial representation of the pipeline of processes, and a summary of all techniques is presented in Table 5.

### 3.2. Machine Learning Methods 

#### 3.2.1. Ischemic Stroke

***CT based methods:***

For CT images, an ischemic stroke appears as a dark or low attenuation (hypodense) region, well contrasted against its surroundings. For early detection, manual processing via a clinical expert has traditionally been the most effective, but it is time–consuming (especially under the time pressures of acute stroke management). Hence, an emphasis is given to automation of detection using machine learning techniques. Rajini et al., developed an approach for segmentation with amalgamation of texture analysis and the midline shift tracing algorithm [65]. Quantifying cerebrospinal fluid (CSF) volumetric changes over time is a potential biomarker for cerebral edema, and these studies performed this by using ML [66,67]. Guberina et al., performed the Alberta stroke program using ML techniques to detect early infarction sign [68]. As the features extracted from MRI yield better results, we see a major use of ML techniques with MRI.

***MRI based methods:***

To date, MRI is the most sensitive modality to detect hyperacute stroke by determining early cellular swelling due to ischemia. Brain ischemia produces effects that are time variant. Hence, dynamic changes are seen in MRI, from early hyperacute (0–6 h of onset) to chronic (≥3 months) staging [69,70]. Teruyuki et al., found that in case of acute stroke, mismatch of abnormalities between images of perfusion–weighted MR and DWI could help identify the penumbral region [71]. Maier et al., presented a comparison study of different ML based classification methods for ischemic stroke lesion segmentation [72]. Mitra et al., explored the probabilistic method of Bayesian–Markov random field (MRF) for segment (FLAIR) MRI and employed random forests (RFs) to extract highly probable lesion areas [73]. Bharathi et al., explored ways to enhance segmentation quality using handcrafted and unsupervised techniques and derived features [74]. Yoo et al., performed a study to determine optimum thresholds of MRI modality parameters to aid the decision to provide reperfusion therapy on the onset of stroke symptoms [75]. Maier et al., proposed an automatic method of extra tree forests for voxel–based classification with an emphasis on reproducibility and robustness to noise [76]. 

Ensemble techniques have been widely employed for better results. Mark et al., applied five ML algorithms (viz. generalized linear, additive model, adaptive boosting, SVM, and RFs) to outline intense cerebral ischemic tissues that can recover after reperfusion [77]. Bagging technique such as RFs have been popular amongst most of the detection work, perhaps due to their high resilience to variance. Muschelli et al., and Qaiser et al., experimented with RF customized features (local moment details, MRI’s scan, smooth and median intensities) to predict the presence of ischemic penumbra and segmentation [78,79]. Fusing and cascading classifiers distributed across reference space and grouped to be classified with high–level region–specific RFs have yielded good results [80]. Hanna et al., presented a technique for segmentation using RFs with context–based clustering techniques [81]. Jerman et al., integrated an unsupervised segmentation technique with RFs (supervised) [82]. Mckinley et al., proposed an automatic method for segmenting ischemic penumbra using spatial and textural features on “Segmentation Forests” [83]. Robben et al., proposed a segmentation technique using cascading extremely randomized forest classifiers [84]. Chen H et al., proposed a segmentation technique using dense conditional random fields to enhance the probability maps which are then used to train RFs [85]. To deal with the issue of model generalization and the inability to be specific to cater to the highly dynamic expressions of pathology, Goetz et al., proposed a methodology which adaptively samples optimal images from a training set to train classifiers (thereby supporting heterogeneous databases) [86]. Few have experimented with other supervised techniques either distance based (k–nearest neighbor) or probabilistic (Gaussian Naïve Bayes) [87,88]. Karthik et al., utilized the discrete curvelet transformation with a few statistical parameters as features on different scales to train the RBF kernel SVM model and ANN [89]. ML models have the tendency to become complex in lieu of better performance. Pereira et al., proposed an unsupervised technique (RBM) for feature learning and to feed the RF classifier for penumbra estimation and evaluation of tumor segmentation [90]. Lin et al., conducted a study to assess quality and identify potentially erroneous measurements due to the presence of outliers. He evaluated and confirmed the suitability of a density–based detection method [91]. Subudhia et al., have used Delaunay triangulation (DT) for optimizing segmentation and tuned the parameters through “Fractional Order Darwinian particle swarm optimization” (FODPSO), for automatic segmentation of stroke lesions [92]. Table 6 contains a summary of these techniques.

#### 3.2.2. Hemorrhagic Strokes

**Figure 8:**
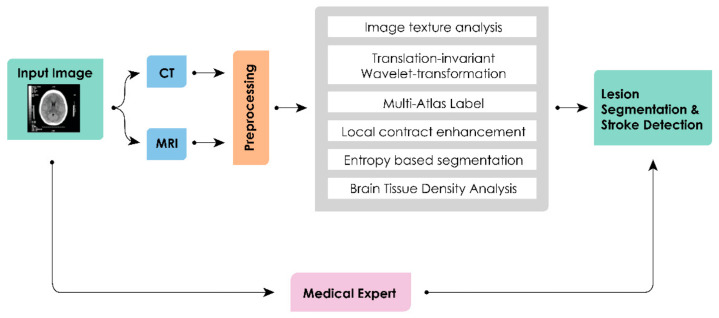
Generalized stages for lesion segmentation, identification, and classification of stroke regions.

Intracranial hemorrhage is defined as bleeding that occurs inside the brain parenchyma. Chen et al., showed an interesting way for detection of brain hemorrhagic diagnosis using Internet of Things [95]. Gillebert et al., present a method to automatically delineate infarct and hemorrhage in stroke CT imagery [96]. The process involves normalized CT images from stroke patients into a template space, and the subsequent voxel–wise comparison with a group of control CT images for defining areas with hypo– or hyper–intense signals. Diagnosing ICH is straight forward. However, identifying early hemorrhagic transformation in ischemic stroke can be challenging. Thrombolysis (fibrinolytic therapy), is the process of breakdown of clots (blood) formed in vessels using medication. This could be lifesaving in case of ischemic stroke but disastrous in cases of hemorrhage. Hence, there must be a method to first identify the stroke before administering thrombolysis. Bentley et al., conducted a study with the ML (SVM) model for predicting the presence of ICH [97]. In Figure 8, the stages for lesion segmentation, identification, and classification of stroke regions for machine learning techniques are shown. Table 6 contains a summary of these techniques.

### 3.3. Deep Learning Methods 

#### 3.3.1. Ischemic Strokes

***CT based methods:***

CNNs are widely useful for adaptability and recent experiments provide evidence of good results using 3D CNN, which captures volumetric information. Chin et al., addressed the difficult task of segmenting acute ischemic lesions, due to their subtle nature as compared with traditional CNN [98]. Identification of highly dynamic texture and intensity variations in pathology is a difficult task using NCCT given the poor visibility. Lisowska et al., investigated the betterment in working accuracy of CNN when appended with spatial information (ATLAS). Although this network performed better due to this incorporation, it was found to be less useful in the case of ischemia [99]. Abulnaga et al., extracted contextual information using a pyramid pooling net (pyramid scene parsing network) [100]. Lucas et al., developed a 3D U–net to predict the final form of lesion with trained clinical knowledge (core and penumbra shapes) represented in lower dimension by a convolutional auto–encoder [101].

Many variants of recurrent networks have been utilized for stroke detection. Vargas et al., built a Res–CNN stacked with a (long short–term memory (LSTM) layer) to check the presence of ischemic stroke [102]. Barman et al., devised a deep symmetry sensitive network (in lines of Siamese networks and inception modules) to analyze symmetrical information [103]. Clèrigues et al., used asymmetric res–encoder–decoder model CT imagery for detecting core infarcts using 2D patches [104]. Shinohara et al., proposed a DCNN model to identify a hyperdense middle cerebral artery (a clinical sign indicating blockage of the artery) to segment regions of ischemic lesion [105]. Due to the wide scale of hypo densities in CT images, it is prudent to utilize an ensemble technique for better generalization and specific results. Barros et al., used three different CNNs for segmentation of subtle, intermediate, and clear hypo–dense lesions. It was seen to be reliable and provided excellent correlation with the reference infarct volume [106]. 

Oman et al., explored the possibility of appending cerebral hemispheric comparison CTA and NCCT as input in addition to CTA to possibly improve the performance of CNN in the detection of AIS. It was reported to have two–fold benefits first being the increased specificity in ischemic lesion detection specificity and second to decrease the number of false positives [107]. Hu et al., proposed a faster, efficient network for lesion segmentation [108]. Islam et al., proposed a training segmentation model using adversarial learning, as this would detect and rectify higher order inconsistencies between the segmentation maps produced by ground–truth and the segmentor. The model consists of a segmentor (generative model) which generates the synthesized model, and a discriminative model that estimates the likelihood of a sample being from ground truth data [109]. Bertels et al., proposed a CNN model with the data present in a nearby (contra–lateral side) voxel for voxel–wise lesion segmentation of the core lesion [110]. Kuang et al., proposed a novel multi–task learning approach i.e., EIS–Net, to segment early Infarct and score “Alberta Stroke Program Early CT Score (ASPECTS)” simultaneously on baseline NCCT scans of AIS patients [111]. Avetisian et al., experimented with altered U–Net CNN architecture by slimming the encoder for the detection of stroke [112]. Robben et al., used a data driven and deconvolution free approach to have a deep learning network to predict the final infract volume [113]. Wang et al., proposed a method to extract features using the RF classifier for automatic stroke lesion 3D segmentation [114].

***MRI based methods:***

MRI, with its inherent excellent soft tissue contrast resolution of the whole brain, offers a simple post–processing operation and provide the flexible ability to simultaneously perform diffusion imaging. However, co–existing MRI findings such as underlying cerebral deep white matter chronic micro–ischemia and indistinct stroke area can sometimes be difficult to segment. CNN have proved to better performing for semantic segmentation [115]. Havaei et al., proposed a CNN based two–pathway framework trained directly on modality, where each path focused on smaller and larger details [116]. Stier et al., built and evaluated a DL model to predict tissue survival outcome based on sampled (randomly) local patches of the hypo–perfusion (Tmax) feature measured immediately after the onset of symptoms [117]. Dou et al., proposed an automatic 3D CNN model for performing a detection operation using a cascading framework [118]. Choi et al., proposed an ensemble of DNNs for the technical tasks of prognosis of post–treatment in case of stroke. This study gave a multiphase learning technique to address the class imbalance problem [119]. As this process is deep and heavily parametrized, and would certainly take a longer time to converge, there are scopes to enhance the computational efficiency and improve time/space constraints.

Although diffusion–weighted MR imaging (DWI) is sensitive to the lesions, manually localizing and quantifying them is costly and challenging in terms of time and resources. Wang et al., proposed an attention–based DNN with synthesized pseudo–DWI from perfusion maps to obtain superior image quality for better segmentation [120]. Chen et al., proposed model framework consists of two CNNs for segmentation (automatic) of DWI based stroke lesions in DWI. The architecture contains an ensemble of two DeconvNets for detection of lesion, followed by a second CNN (MUSCLE Net) for refinement and identifying and removing false positives [121]. Lucas et al., studied the use of classical fully–connected neural networks (FC–NN) (151 features) based on handcrafted featuring, and compared the results with DCNN and RF models in terms of accuracy and convergence time. FCNN achieved much shorter runtimes [122]. Alex et al., proposed a de–noising auto–encoder model for unsupervised feature learning of brain lesion detection, segmentation, and reducing false positives [123]. Giacalone et al., employed the local spatial information (temporal) for prediction of final lesion [124]. Perfusion imaging is essential to assess penumbra area and infarcted core due to its ability to measure blood flow, transition times and dispersion. Lucas et al., implemented an extension of U–Net, and added skip connections after an alternative 3x3 Conv Block. Surface distance proved to be more useful than pixel/voxel matching for irregular shape and to avoid low scores [125]. Bento et al., performed a study and build an architecture for identification of atherosclerosis areas [126]. Song et al., proposed a novel generative technique consisting of extractor (features from CTP), generators (DWI based features) and segmentor [127]. Liu et al., proposed a 2D–slice–based segmentation method with a residual–structured FCN (Res–FCN) on the multi–spectral MRI process. Many blocks of CNN were involved for better feature extraction [128]. Zhang et al., proposed a deep 3D CNN for automatic segmentation by extended DenseNets to 3D and tapped their potential on AIS segmentation from DWI. They employed a Deep supervision technique and Dice objective function to improve optimization [129].

Chen et al., proposed a novel voxel–wise residual network (VoxResNet) with a set of effective training schemes to address segmentation in the complicated anatomical environment of the brain and the large variations of brain tissue [130]. Li et al., presented a 2D ensemble FCNN based architecture to spot hyper–intense regions in fluid attenuated inverse recovery (FLAIR) and T2 weighted imagery [131]. It is seen to achieve best results on hand crafted features, which in turn are complex and often lack the ability to distinguish between affected and normal tissue. Praveen et al., proposed a stacked sparse auto encoder framework for automatically learning and selecting features followed by the SVM classifier to accurately segment stroke lesions from brain MR images [132]. Due to the limited number of labelled and high–resolution scans, currently many investigators generate synthetic data and train the model adversely. Alex et al., proposed a semi–supervised technique with a generative adversarial network (GAN) for brain lesion segmentation [133]. Li et al., proposed an 2D dilated deep residual network to capture contextual information for segmentation task [134]. Luna et al., proposed a novel 3D CNN based with transition layers between encoding and decoding process to increase the impact of features maps in latter phase [135]. Winzeck et al., investigated whether an ensemble of convolutional neural networks trained on a multi–parametric DWI (MRI) mapping outperforms single networks trained on solo DWI parametric maps [136]. Liu et al., proposed a DCCN (Res–CNN) to automatically segment acute ischemic stroke area from multi–modality MRIs. In contrast to the single modality version, use of multimodality helps to improve segmentation performance [137]. Karthik et al., proposed a supervised DFCN, with leaky ReLU as the activation in the last two layers of the network for a precise reconstruction (absent in U–Nets) [138]. Li et al., presented a unique end–to–end brain tumor segmentation method by modifying the up–skip connection between the encoder and decoder, and adopting the inception module (7×7 high receptor convolutional layers) in each block to help the network learn richer representations [139]. Malla et al., explored the scope to evaluate the impact of enhanced ML techniques, advancements, transfer learning, and post–processing in the segmentation of stroke areas. [140].

These studies underscored the importance of multi-scale features and contextual features and ways to capture long range dependencies [141,142]. Liu et al., proposed a DCNN for stroke MRI based segmentation to address overfitting [143]. Chin et al., showed that ensemble techniques like cascading could be used for post stroke analysis to improve results [144]. Studies have been performed to perform segmentation using neighborhood or symmetry information [145,146]. Rajan et al., proposed adversarial trained res–net model to showcase the effectiveness of a boundary weighted loss function [147]. Lui et al., improved the performance of segmentation using attention mechanism [148]. Zhang et al., proposed multi-plane fusion architecture for stroke segmentation [149]. Amin et al., employed a high pass filter image to make prominent the in–homogeneity field effect of the MR slicing, and fused it with the input slices [150]. Bui et al., proposed a novel fully automatic Dense Net (adversarially trained) for predicting volumetric probability maps [151]. Xue et al., proposed a multi–modal multi–path convolutional neural network system for automating stroke area segmentation by analyzing brain–behavior relationships, thereby eliminating the need for manual segmentation. Joshi et al., proposed an encoder–decoder CNN (dilated) for an ischemic lesion segmentation task; this helped in preventing data loss which can occur during max–pooling [152].

**Figure 9:**
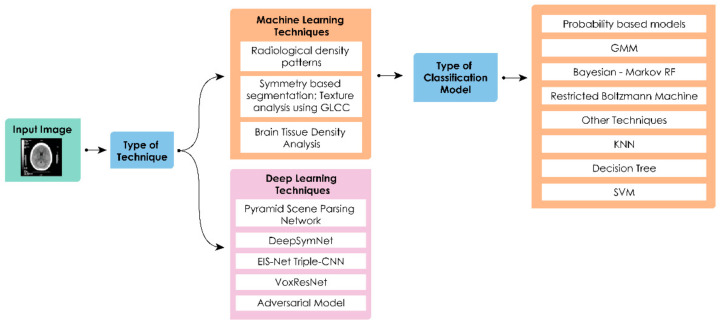
Generalized stages for lesion segmentation, identification, and classification of stroke regions.

The penumbra is the area surrounding an ischemic event, which can be salvaged if prompt treatment is received. Delineation of the penumbra in relation to the infarcted core is important for stroke treatment and monitoring the treatment success. Gupta et al., proposed a multi–sequence network for the segmentation of ischemic lesions and to differentiate between core and penumbra. Both the core and penumbra sequences are fed into a U–Net type network [153]. Kumar et al., proposed DeepNet framework for ischemic segmentation [154]. Satish et al., presented an automatic method for identification of core and penumbra regions in ischemic lesions using DWI and perfusion–weighted imaging (PWI). In the absence of the availability of more labeled data, the CNN is trained adversarially (i.e., synthesizing images, applying a segmentation loss (cross–entropy)), with aggregated losses from three discriminators (two of which have the relativistic visual Turing test) [155]. Figure 9 shows the stages for lesion segmentation, identification, and classification of stroke regions for deep learning techniques. Table 7 contains a summary of these techniques.

#### 3.3.2. Hemorrhagic Stroke

Phong et al., compared three types of CNN: LeNet, GoogleLeNet, and Inception ResNet to determine the best method for hemorrhagic stroke detection [156]. Majumdar et al., trained a CNN with improved performance by computing the mean output for rotations of input images [157]. Arbabshirani et al., proposed and tested a predictive DL model capable of detecting ICH [158]. Kuo et al., dealt with a challenge to identify minute and subtle abnormality in a large 3D volume with superior sensitivity, through an end–to–end patch–based FCN model network that performs joint classification and segmentation on CT images [159]. Patel et al., proposed a 3D CNN with a combination of contextual information to detect and segment stroke lesions [160]. Cho et al., proposed a deep learning model that was constructed on two convolutional neural networks and dual FCN to detect bleeding, for classification into five types of ICH for lesion segmentation [161]. The limited hardware poses a problem in computation for deep learning networks, and there exists a tradeoff between hardware and input size (i.e., learning via contextual information). Patel et al., tried a method for the identification of ICH in 3D NCCT. The method combines a CNN and RNN through bidirectional long short–term memory (LSTM) for ICH identification at the image level [162]. Barros et al., proposed and developed CNN for the detection and volumetric segmentation of subarachnoid hemorrhage (SAH) in non–contrast computed tomography (NCCT) [163]. Lee et al., trained a DL model for detection of ICH without backpropagation [164]. Xu et al., introduced the continuous monitoring of health vitals wirelessly through IoT, which is termed ‘Health of Things’. The system is capable of classifying CT imagery into uninjured and stroke and thereafter, the segmentation process is carried out via a combination of Masked RNN and ML algorithm [165]. Li et al., proposed a U–net based DL framework to detect and segment hemorrhagic strokes based automatically on CT brain images.

Experiments are conducted to add a symmetrical constraint by using flipped images as input [166]. Arab et al., developed and evaluated an automated DL method with CNN and deep supervision CNN for precise hematoma (blood clot) segmentation and volumetric quantification in CT images [167]. The combination of CNN with LSTM has yielded good results but shows limited accuracy in performance, as many types use pre–trained models. Grewal et al., proposed a RADNET joint CNN and LSTM model which emulates a radiologist for ICH detection, by performing segmentation at multiple levels of granularity and including a binary classification of intracranial hemorrhage [168]. Burduja et al., proposed a light and efficient network for detecting ICH, consisting of the CNN and LSTM [169]. A summarized review with details is highlighted in Table 8.

#### 3.3.3. Combined Stroke

Pereira et al., present a model for stroke detection in CT using CNN optimized by PSO; the shallower network obtained better accuracy than the deeper version [170]. Marbun et al., employed CNN with proper preprocessing of images (gray scaling, histogram equalization etc.) for classification of type of stroke [171]. Carlos CMD et al., presented an IoT enabled framework with CNN as the main classifier to identify a healthy or a stroke affected brain from CT images [172]. Kunag et al., proposed to segment ischemic and hemorrhagic infarct simultaneously using a U–Net based architecture. The input is divided into four disjoint regions and CNN was employed to generate probability maps for ischemic, hemorrhagic, and other infarcts [173]. Xuea et al., used multi–modal MRI for classification of types of strokes [174]. 

## 4. Discussion

### 4.1. Non–ML/DL Based Techniques

Several papers were surveyed under this topic to understand various approaches and techniques. It was found that all of these basically fall under several major categories: region growth, texture extraction (linear, non–linear, and spatial and frequency domain), enhancement and analysis and contour based. Time complexity was largely governed by modality type in consideration with the degree of imaging modals. Although many algorithms have automated the processes, much of this work still requires manual intervention and supervision. Work towards consideration of data encompassing various other parameters such as age and essential clinical parameters could be helpful in making a more comprehensive analysis.

### 4.2. ML Based Techniques

Publications in this domain may be classified into three major types: (1) classification based on discriminants such as texture, brain tissue density, contour–based analysis; (2) purely probabilistic types; and (3) hybrid types, where the results are refined by other methods. Although the former is efficient in terms of reducing time complexity and processing, they suffer from the need of specialist intervention to indicate region and to affirm the stroke type. Furthermore, for effective demarcation there is a need for optimal radiation attenuation, which poses as considerable challenge. Most of the texture–based algorithms have the drawback of missing barely visible lesions due to subtle intensity differences, scan image quality, and intensity inhomogeneity, which causes high false positive rates. A system with medical expert assistance to aid in the technical detection could prove helpful for network training [175]. It is therefore expected that future research directions will include the development of intensity resilient algorithms which perhaps could be coupled with better image enhancement techniques.

### 4.3. DL Based Techniques

Emergence of deep learning has established a new paradigm in the domain of stroke detection. Many papers have shown remarkable progress in terms of time, accuracy, and adaptability. Their ability to customize feature importance and to identify discriminating features including the presence of hyperdense vessels (sign of a large vessel occlusion), and disturbed symmetry of vascular and brain tissue textures, has been primarily used for prognosis and automatic detection of lesions. Architectures such as CNN with 3D kernels have been extensively explored with many modifications in loss functions (specialized focus) and adaptations to novel model architectures (U–Nets, ResNets, etc.) have led to improved efficiency. However, CNN architectures are limited in accuracy when segmenting ischemic stroke areas, and their heterogeneity in location, shape, size, image intensity, and texture are the main reasons for their reduced level of efficacy, especially in this imaging modality. Although such techniques rely on self–extraction of features, it was found that many yielded better results after providing additional information including the use of atlas coordinates to show dependency. In CNN architectures, the kernel size determines the ROI, thereby affecting diagnostic performance. Smaller kernels lead to missing specific regions and bigger kernels lead to heavy parametrization. Hence, a tradeoff is imperative. Most of the considered datasets seem to be highly imbalanced leading to overfitting; this could be solved by incorporating precision or recall based upon an objective function with data fusion [175]. Moreover, data augmentation techniques and generation of synthetic data using GAN may need to be utilized. We found a lesser number of papers dedicated to the delineation of core and penumbra regions separately. Hence, further research should be extended in that direction. 

### 4.4. Preferred Choice of Diagnostic Imaging

There is no single globally preferred choice in modality either for lesion separation or stroke detection; all have their benefits and are specific to certain tasks. In our study we found that there is an urgent need for better imaging analysis technology to improve inference, and a need for advancements in imaging techniques, as detection of incomplete infarction in the acute stroke setting on MRI or CT is currently not feasible (since current CT and MRI modalities are subjected to partial volume averaging as well as very limited spatial and tissue resolution). Yet in practical scenarios, accessibility must be the primary concern for such applications. As CT is readily accessible, affordable, and less contraindicated, especially for hemorrhagic stroke analysis, it is seen as a natural choice for implementation [175]. In most of the analyses we found that CT is useful in many applications, providing acceptable results and often the best results. We noticed many papers which employed raw CT as their base modality. In advanced cases, perfusion CT addresses many critical unknowns in the acute stroke triage, and it is apt in the task of delineating the operational penumbra from the infarct core. In application of acute intracranial ischemic/hemorrhage NCCT this was seen as extensively useful.

More work should be carried out in the advancement of CT or its variants in application of networks to explore the core as a potential area to improve stroke analysis. We do not claim that CT is better than MRI for this purpose, but from a practical standpoint there is reason to enhance the processing of CT data for preliminary diagnostics and prior to the use of higher advanced modalities in following stages.

### 4.5. Time Complexity

Time complexity is a measure of the time consumption of algorithms as a function of inputs, preferably measured for the worst–case scenario to set the upper limit [176]. Although it is an important metric for gauging algorithms, it is tenuous when used for complex domains such as segmentation, detection, and prediction of intricate regions, as they are often an amalgamation of many techniques working either sequentially or in parallel. In this review, few studies have reported the time taken for processing per input or relative improvement in terms of time while also providing a detailed analysis of their algorithm in terms of time and space complexity. Including a section on “computational and space complexity” of their model can be one of the evaluation parameters used to assess the performance of the model. 

### 4.6. Prognosis

The ultimate goal of ischemic stroke treatment is to recanalize an occluded vessel and enable damage control. It is important for timely and precise decision–making, which ultimately affects patient outcomes. This study attended to the detail of stroke prognosis, plausibility of treatment options, and relevance. We studied many research papers which performed and successfully executed analysis to aid in diagnosis and prognosis. A summary is illustrated in Table 9. There were many dimensions that investigators used such as prediction of infarct growth over time and predicting the functional outcome of ischemic stroke patients, but these were limited by insufficient data, lack of manual supervision, and massive size. As many studies have pointed out, the most probable reason behind stroke is older age, lifestyle, low level of physical activity, unhealthy diet, hypertension, smoking, and diabetes mellitus, which makes the prognosis based on a specific source difficult. In this regard, we wish to encourage the research community to explore the areas of personalized diagnostics with many sources of consideration. Deep learning models utilizing image features coupled with other information about the patient could yield better and more accurate results. The capturing of data can be carried out via a home medical teleport, or from nearby health centers, and then transmitted to cloud–based models via mobile apps, even in remote regions of the world with lesser available services. Cloud–based models trained and maintained using federated learning are potentially much more reliable than current methods and could revolutionize this field.

## 5. Challenges and Future Directions

While surveying the field, we encountered many approaches, applications, and techniques based on various datasets, and it was difficult to evaluate them all in generality. Many methods claimed to be fully automated yet relied on human assistance/interaction for parameter initialization. A fully automated process would require a powerful intelligent system which can adapt and customize based on patient condition/severity of symptoms and would avail a host of opportunities regarding the prospects of artificial intelligence in this area. A remarkable progress in terms of segmentation has already been carried out, yet the task of specifically locating the penumbra and core is still to be explored and refined. Concerning identification of the penumbra, more work is needed as this would help to rejuvenate it faster. In stroke detection, we found less work in identification of its sub–classes and a lack of research on the dynamic evolution of stroke as time progresses. A heterogeneous dataset containing images across the regions and countries with different conditions must be developed for better research and more clarity on their impact.

**Figure 10:**
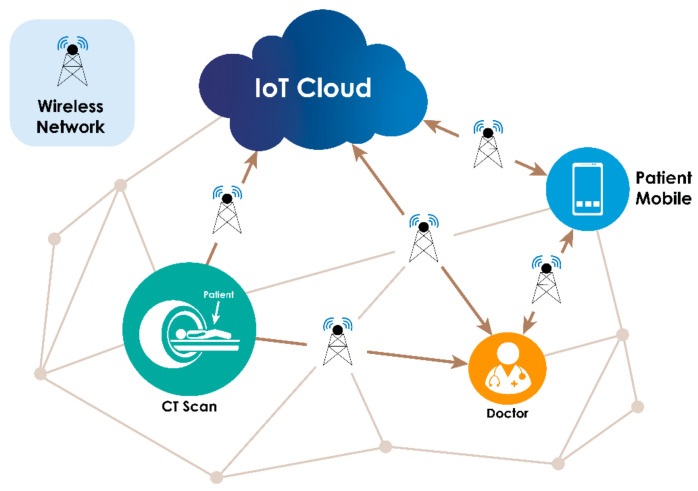
Prototype model for remote patient monitoring with cloud–based AI Model.

Future research can be directed in several ways:

1. IoT based personalized AI: AI being the main protagonist of Industry 4.0, having far–reaching implications, especially in healthcare. Hyper personalization of healthcare could provide tailor–made diagnostics and would vastly improve early detection of disease.
2. Creation of a large hetero public database: The dataset that we have addressed consists of few images for train and test, with regard to particular domain or region. A larger public dataset would assist to better cover major areas.
3. Remote patient monitoring through federated learning: Figure 10 shows a prototype for remote patient monitoring with cloud–based AI Models. Wearable modes with continuous monitoring of biomarkers with easy transfer of meta–data to cloud through phones for collective learning and personalized prediction would be helpful. These could act as a digital expert to assist in patient diagnosis and prognosis.

## 6. Conclusions

In this study we reviewed the status, trends, and future directions in stroke detection and segmentation. It is clear that a rapid, adaptable process facilitating timely neuroimaging analysis is imperative in stroke management. This is due to the fact that neuroimaging has a prime role in the diagnosis and optimal management of different types of strokes. Due to recent advancements in neuroimaging, AI, and computation power, the development of automated diagnostic tools is clearly within reach. Advances in this field and translation into clinical practice will result in reduced patient morbidity and mortality.

Based on the findings of this systematic literature review, we make a number of suggestions for how the performance of automated diagnostic tools can be improved and a more comprehensive automated system can be built. Firstly, segmentation techniques could be automated and personalized for individual patients, allowing translation of clinical research into clinical diagnostic practice. Secondly, instead of training the systems on specific datasets, a more heterogeneous dataset could be considered. This would help the models to comprehensively learn all cases, both regionally and country–wide. Thirdly, besides the prospects mentioned on the different stroke stages in Section 5, a fully automated segmentation system with deeper networks for stroke area segmentation (especially in sub–acute and chronic strokes) could be built in for future paradigms. Fourthly, as there have been recent attempts to predict infarcts and the extent of the penumbra, more research should be focused on these strategies, in order to devise and combine prognostic tools for strokes, such as the degree of infraction, into stroke management algorithms. Fifthly, GANs could certainly prove helpful in generating synthetic datasets in cases of data scarcity, class imbalance and cases when the cost of obtaining labelled data is huge. However, care must be taken to keep original distribution unperturbed and that new data doesn’t create any bias in the decision-making process. Sixthly, segmentation models greatly depend on quality of image quality, acquisition and the reconstruction parameters of the modality. Small changes in these parameters can lead to a substantial deviation in the output in model output [177]. It could be better to have proper standards for parameters can which could potentially prevent this and help in improving the reproducibility of the results. Lastly, it would be ideal to explore the possibility of remote patient monitoring, to enhance equity of access to excellent stroke management in the most cost–effective and acceptable manner, and also boost the prediction of stroke, with the potential to prevent this disabling condition arising in the first place, thereby trans–forming patient outcome. Perhaps in the future, the accuracy of stroke prediction via metadata analysis will be an important criterion to evaluate stroke segmentation results.

## Figures and Tables

**Table 1:** Datasets and Benchmarks.

Modality	Database	Data Size	Area	Classes	Ground Truth	Data Info
MRI	ISLES 2015	**SISS**: 28(train) 36(test) **SPES**: 30(train) 20(test)	**SISS**: sub–acute ischemic stroke lesion segmentation **SPES**: acute stroke outcome/penumbra estimation	**SISS**: Lesions were classified as sub–acute infarct and Infarct lesions. **SPES**: target mismatch = perfusion–restriction label minus diffusion–restriction label	SISS: Segmentation by an Expert	http://www.isles-challenge.org/ISLES2015 (accessed on 4 October 2021).
MRI	ISLES 2016	35(train) 19(test)	Dataset provides a regression and segmentation and a task: **Task 1**: prediction of Lesion outcome **Task 2**: prediction of Clinical outcome	MODIFIED RANKIN SCALE (MRS) The 90 days mRS is a scale to assess the degree of disability 90 days after a stroke incidence (Task II assessment) (Grade: G) G 0 No Symptoms. G 1 No significant disability despite symptoms; G 2 Slight disability; need assistance G 3 Moderate disability G 4 Moderately severe disability; G 5 Severe disability; G 6 Dead.	Final lesion volume (Task 1) as manually and the clinical mRM score (Task 2) denoting the extent of disability	http://www.isles-challenge.org/ISLES2016/ Highest **I**_DC_ = **3.37**
MRI– (DWI, ADC)	ISLES 2017	43(train) 32(test)	Acute ischemic stroke (Challenge for stroke lesions segmentation, core and penumbra separation)		Ground–truth segmentation maps manually drawn on scans	Lesion outcome (prediction) based on acute MRI data. http://www.isles-challenge.org/ISLES2017/ Highest **I**_DC_ = **4.53**
CBF, MTT, CBV, TMAX, CTP	ISLES 2018	63(train) 40(test)	Penumbra–core separation using CT		Expert segmentations of the infarct lesions.	Acute ischemic stroke patients with 8 hrs. of stroke onset and MRI DWI within 3 h. after CTP. http://www.isles-challenge.org/

**Table 2:** The exclusion and inclusion criteria.

Inclusion	Exclusion
Studies pertaining to	Studies pertaining to
1. CT and MRI (including variants)	1. Treatment of strokes (Exclusively)
2. Ischemic and hemorrhagic strokes	2. Pure Statistical and Biological methods of treatment.
3. Measurement of the degree of the infarct and damage.	3. Technical working and advancement of algorithms
4. Prognosis of strokes and the likelihood of damage	4. Lesions extraneous to strokes
5. Lesion detection and segmentation (core and penumbra region)	
6. ML and DL techniques for segmentation of lesion regions	
7. Latest architectures in DL techniques and factorization techniques for feature–specific algorithms.	

**Table 3:** Modalities at a Glance.

Modality	Description
NCCT (CT)	CT uses a beam of X–rays followed by a process of high–powered computers to generate images of soft tissues and bones. Overall sensitivity of 57–71% and 12% in the first 24 h, 3 h respectively [31,32]
Perfusion CT	These scans help identify areas adequately supplied with blood (perfused) and provide detailed information about blood flow to the brain. Regions which demonstrate matched defects in MTT and CBV represent the unsalvageable infarct core, whereas regions with prolonged MTT, but preserved CBV are considered to be the ischemic penumbra, and are potentially salvageable [32]
Angiography CT	CT angiography is a type of medical test that combines a CT scan with an injection of a special dye to produce pictures of blood vessels and tissues. Within an intracranial vessel it may also identify thrombus, and may guide for intra–arterial thrombolysis or clot retrieval [32]
MRI	MRI is based on the magnetization properties of atomic nuclei. Protons in the water nuclei of tissues are excited and relaxed, and subsequently capturing the released energy. Based on the relaxation time, T1 and T2 tissues are characterized [32].
T1 weighted (MRI)	Characterized by shorter relaxation time. Following noticeable changes in scans [32] CSF appears dark 2. White matter appears light 3. Cortex appears gray 4. Inflammation appears dark
T2 weighted (MRI)	Characterized by longer relaxation time. Following noticeable changes in scans [32] CSF appears bright 2. White matter appears dark gray 3. Cortex appears light gray 4. Inflammation appear bright
Flair (MRI)	Characterized by longer relaxation time than T2 weighted images. Following noticeable changes in scans [32] CSF appears dark 2. White Matter appears dark gray 3. Cortex appears light gray 4. Inflammation appears bright
DWI (MRI)	Detect the random movements of water protons. Spontaneous movements, rapidly become restricted in ischemic brain tissue which appear bright in scans. It is an extremely sensitive method for detecting acute stroke. [32] Apparent diffusion coefficient (ADC) is a measure of the magnitude of diffusion (of water molecules) within tissue. Rough values (10^−6^ mm^2^/s): CSF: 3000–3400 2. White matter: 670–800 3. Cortex: 800–1000

CSF, cerebral spinal fluid; CBV, cerebral blood volume; MTT, mean transit time.

**Table 4:** Radiological features based on modalities.

Stroke	Ischemic			Hemorrhagic		
Modality	Acute (0–7 days)	Subacute (1–3 Weeks)	Chronic (>3 Weeks)	Acute (0–7 Days)	Subacute (1–3 Weeks)	Chronic (>3 Weeks)
**NCCT**	Loss of grey–white matter differentiation, and hypo attenuation (low density, obstruction) of deep nuclei [31,32]	Attenuation of the cortex [31,32]	Hypo density region [31,32]	Hyper dense with fluid levels [33]	Less intense with ring–like profile [33]	Iso dense or modest confined hypo density [33]
**T1**	Low T1 signal [32]	Low T1 signal [32]	Low T1 signal [32]	Iso intensity or slight hypo intensity with thin hyper intense rim in the periphery [32,33]	Hyper intensity [32,33]	Hypo intensity [32,33]
**T2**	infarct remains Hyper intense [32]	Hyper intensity [32]	High T2 signal [32]	Hypo intense with hyper intense perilesional rim [32,33]	Hyper intensity [32,33]	Hypo intensity [32]
**DWI**	Decreased ADC values with maximal signal reduction within 1 to 4 days marked with hyper intensity [32]	First ADC values rise and return close to baseline, despite normal ADC values irreversible tissue necrosis is present (DWI remains hyper intense) [32]	ADC signal high [32]	ADC: 0.70 [35]	ADC: 0.72 [35]	ADC: 2.56 [35]

ADC, apparent diffusion coefficient.

**Table 5:** Summary of computer aided statistical techniques for lesion segmentation and stroke detection.

Articles	Modality	Technique	Outcome	Year
Tang et al. [38]	CT	Image texture analysis through Circular Adaptive Region of Interest method	S_ROC_: [0.99–0.94]	2011
Sajjadi et al. [39]	CT	Translation–invariant wavelet for image enhancement	Higher information image extracted	2011
Nowinski et al. [40]	NCCT	Analyzing hemisphere attenuation values using percentile difference ratios	**S**_Acc_ is 83.2%. The early detection accuracy (<3 h) is 78.4%.	2013
Filho et al. [41]	CT	Analysis of brain tissue density		2017
Flottman et al. [42]	CT	Novel threshold free method		2017
Lo et al. [44]	NCCT	Local contract enhancement using Ranklet Transformation and probability based detection	GLCM Ranklet **S**_ACC_ 71% 81%	2019
Bhaduria et al. [48]	CT	Segmenting through the features of both fuzzy clustering and region–based active contour model	**S**_DC_: 0.92	2014
Haan et al. [49]	CT, DWI, T2FLAIR	Clustering algorithm for lesion demarcation in AIS	Reduced processing **time** to on average 17.8 min/patient	2015
YAHIAOUI et al. [50]	CT	Differentiation of brain pathology area (hypodense) from its adjacent normal parenchym (i.e., contrast enhancement) using Laplacian Pyramid	Laplacian Pyramid algorithm gives Better and faster (10.46 s) result than DWT, especially in small sized lesions.	2016
Reboucas et al. [51]	CT	Level set based approach on brain densities (radiological) method to generate stroke segmentation	Segmentation time and S_ACC_ LSBRD (proposed) 1.76, 99% Watershed 3.10, 92% Region Growing 4.81, 93%	2017
Kumar et al. [52]	CT	Entropy based segmentation	**S**_ACC_: 99.87 (avg)	2020
Vasconcelos et al. [53]	CT	Adaptive Brain Tissue Density Analysis	**C**_ACC_: 98.13%	2020
Nabizadeh et al. [54]	MRI	Histogram–based gravitational optimization algorithm	**S**_ACC_: 91.5%(strokes)	2014
Ghosh et al. [55]		Hierarchical Region Splitting, Symmetry Integrated Region Growing and Modified Watershed Segmentation		2014
Ledig et al. [57]	MRI	Refinement using Multi–Atlas Label based context with Expectation–Maximization.	64.7% **S**_ACC_ using acute–phase	2015
Farsani et al. [58]	MRI	Diffusion restricted characterisitics	**C**_ACC_: 73%	2016
Moeskops et al. [59]	MRI	CNN	**S**_DC_: 0.84–0.91	2017
Ji et al. [63]	MRI	Gaussian Mixture Model	**S**_ACC_: 5% more than baseline model	2017
Kamnitsas et al. [64]	MRI	A 11 layered dual pathway architecture for joint processing of adjacent image patches (DeepMedic)	**S**_DC_ on training data of BRATS 2015 DeepMedic + CRF 89 .8 DeepMedic 89 .7	2017

**S_ROC_**, receiver output receiver; GLCM, gray–level co–occurrence matrix; **S_ACC_**, segmentation accuracy; **S_DC_**, segmentation dice coefficient, **C_ACC_**, classification accuracy.

**Table 6:** Summary of various ML techniques applied for stroke detection (ischemic) and segmentation.

Articles	Modality	Technique	Outcome	Year
Filho et al. [41]	CT	Feature extraction based on density patterns (radiological) and classification of strokes through Bayesian, SVM, kNN, MLP, and OPF classifiers	Fastest extraction time **I_ACC_**: 99.30%	2017
Rajini et al. [65]	CT	Symmetry (mid line shift) based segmentation; image texture analysis using GLCC and classification using SVM, k–NN, ANN, decision tree	SVM **I_ACC_**: 98%	2013
Maier et al. [72]	MRI	Generalized Linear Models, RFs and CNN are evaluated and compared with each other for sub–acute ischemic stroke patients	AdaBoost **I_DC_**: 0.69	2015
Mitra et al. [73]	FLAIR MRI	Bayesian–Markov Random Field and RF	**I_DC_**: 0.60 ± 0.12	2014
Bharathi et al. [74]	MRI T1, T2, DWI and FLAIR	Feature Extraction using GLCM and unsupervised extraction Kmeans clustering; and training RF classifier for detection of ischemic stroke lesion	**I_DC:_** 0.88 **I_ACC_** 0.82	2019
Maier et al. [76]	T1w, T2w, FLAIR and DWI	Extra Tree Forest framework for voxel–wise classification	**I_DC_**: 0.65 ± 0.18	2015
McKinley et al. [83]	MRI T1, T2	Spatial Random Forest	ISLES (leave one out) **I_DC_**: 0.85 (±0.06)	2015
Robben et al. [84]	T1w– and T2w, Flair and DWI	cascaded extremely randomized trees	**I_DC_** SISS 0.57 ± 0.28 SPES 0.82 ± 0.07	2016
Chen et al. [85]	MRI	random forests (cascaded) with dense conditional randomfields	ISLES 2015/BRATS 2018 **I_DC_** of 0.51 ± 0.29/0.86	2020
Griffanti et al. [87]	T2 and FLAIR	k–nearest neighbor	**I_ICC_**: 0.99	2016
Griffis et al. [88]	T1	Gaussian naïve Bayes	**I_DC_** 0.66	2016
Karthik et al. [89]	MRI	Multidirectional features based on Discrete curvelet transform and watershed algorithm for fetching the ROI and then applying support vector machines to develop the classification system.	**I_ACC_** 99.1%	2017
Pereira et al. [90]	MRI	Unsupervised feature learning through RBM with RF classifier	**I_DC_** 0.81 ± 0.84	2018
Lin et al. [91]	CT	DBSCAN, hierarchical DBSCAN (HDBSCAN) and local outlier factor (LOF) for identification of erroneous stroke detection	DBSCAN (Avg) **I_ACC_** 96.9	2019
Subudhia et al. [92]	MRI	Delaunay triangulation based segmentation optimized by Darwinian particle swarm optimization	**I_ACC_** of 0.95	2018
Peixoto et al. [93]	CT	SCM, SVM, MLP	**I_SPEC_** = 99.1%[highest]	2018
Garg et al. [94]	Electronic Data (NLP)	Classification of Ischemic Stroke Subtype (TOAST) using ML (RF, GBM, KNN, XGBOOST, SVM, Extra Trees) and NLP	Kappa stacking: combined data = 0.57	2019

GLCM, gray–level co–occurrence matrix; DC, dice coefficient; **I_DC_**, ischemic stroke dice coefficient; **I_ACC_**, accuracy; **I_ICC_**, intra class correlation coefficient; **I_PREC_**, ischemic precision; **I_FS_**, ischemic FScore; **I_SPEC_**, ischemic specificity; **I_SENS_**, ischemic sensitivity.

**Table 7:** Summary of various DL methods applied for IS detection and segmentation.

Articles	Modality	Technique	Loss Function	Outcome	Year
Lisowska et al. [99]	NCCT	Bilateral CNN + Atlas	squared hinge loss	**I_AUC_:** 0.964	2020
Abulnaga et al. [100]	CTP	Pyramid Scene Parsing Network	Focal Loss	**I_DC:_**0.54 ± 0.009	2017
Vargas et al. [102]	CTP	CNN LSTM [Train 356, Validation 40]		**I_ACC:_** 87.5%	2018
Barman et al. [103]	CT A	DeepSymNet Two identical CNNs with 3 Inception module for learning the low and high level volume 3D representation common to the two brain hemispheres.	L–1 difference	**I_AUC_:** 91.4%	2019
Clèrigues et al. [104]	CT, CT–PWI CBF, CBV& MTT	DL based segmentation approach using 2D patch based for of the acute stroke lesion core.	To minimize the effects of class imbalance Generalized Dice Loss (GDL) with the cross entropy loss.	**I_DSC_** improvement of 4.5% over the baseline [ISLES 2018]	2019
Shinohara et al. [105]	NCCT	Xception architecture pre–trained on the ImageNet database	classification loss	**I_SPEC_**: 89.7% **I_ACC:_** 86.5%	2020
Barros et al. [106]	NCCT	CNN with two convolutional layers (256 nodes, 64/128 feature resp.) followed by 2 FCN. Each dense layer has. Max–polling layer with a 2 × 2 kernel and a 2 × 2 stride.		Severe **I_ACC_:** 0.98 Intermediate **I_ACC_:** 0.93 Subtle **I_ACC_:** 0.66	2019
Oman et al. [107]	CTA, NCCT	3D CNN		**I_DC_:** 0.61	2019
Hu et al. [108]	3D MRI	3D residual framework	Focal Loss	BRATS 2015 **I_DC_:** 0.86 (whole)	2020
Bertels et al. [110]	CTP	Contra Lateral Information CNN	Binary cross–entropy	**I_DC_:** 0.45 [ISLES 2018]	2018
Kuang et al. [111]	NCCT	EIS–Net Triple–CNN with three triple encoders and one de–coder with multi–level attention gate modules.	Combination of weighted binary cross entropy and Generalized Dice–Coefficient.	**I_ACC_:** EIS–Net 85.7%	2021
Avetisian et al. [112]	NCCT	Dual Path Network which fusing the features of Res–Nets and densely–connected networks	Focal Loss	**I_DC_:** 0.703	2020
Wang et al. [114]	MRI	3D RF trained on ISLES dataset	Hybrid loss function	**I_DC_:** 0.16 ± 0.31 [test]	2016
Havaei M [116]	T1, T2, T1C and Flair	CNN (two pathways cascaded architecture)	cross–entropy loss	SISS **I_DC_:** 0.69 SPES **I_DC_:** 0.85	2020
Chen et al. [121]	DWI	CNN	Cross Entropy	**I_DC_**: 0.67 [avg]	2016
Lucas et al. [121]	FLAIR, DWI, T1, and T2	FCNN–MatConvNet	cross–entropy loss	**I_DC_:** 0.59	2017
Alex et al. [123]	T1, T2,T1C FLAIR	Stacked denoising autoencoders		High and Low Grade Glioma	2017
Lucas et al. [125]	MRI	Res–UNets	Weighted sum of a classification and soft QDice metric	33% lower surface distance than U–Net	2017
Liu et al. [128]	MRI	FCN (Res–FCN)	Customized Loss Function	**I_DC_**: 0.645	2018
Zhang et al. [129]	DWI	3D FC–DenseNet	Customized Loss function + Dice Loss function	**I_DC_**: 0.79 [Best]	2018
Chen et al. [130]	3D MRI	VoxResNet: Stacked residual modules with convolutional/de–convolutional (total 25 volumetric)	spatial information loss	**I_DC_** GM 86.15 WM 89.46 CSF 84.25	2018
Li et al. [131]	MRI FLAIR	Two convolutional layers are repeatedly employed, each with ReLU and a 2 × 2 (max pooling), down–sampling with stride 2	Dice Loss	MICCAI 2017 **I_DSC_**: 0.80	2018
Praveen et al. [132]	FLAIR, DWI, T1, and T2	Stacked Sparse autoencoder layers and support vector machine classifier as the output layer.	Mean Squared Loss	ISLES 2015 **I_DC_**: 0.943 ± 0.057	2018
Li et al. [134]	CT, DPWI, CBF	Deep Residual Dilated U-Net	Cross–entropy loss	MICCAI **I_DC:_** 0.81	2018
Luna et al. [135]	MRI	3D CNN	normalized categorical cross entropy loss	MRBrainS18 Weighted DC 4.44	2019
Winzeck et al. [136]	MRI	Ensemble Res–CNN:	Costumed Loss Function	**I_DC_:** 82.2%	2019
Li et al. [139]	T1, T2, T1c and FLAIR	U–Net structure with a new cross–layer architecture (up skip connection) and incorporating inception modules	DSC	[train] **I_DC_:** BRATS 15 0.89 BRATS 17 0.876	2018
Malla et al. [140]	MRI	CNN [Deepmedic]	Dice Similarity Coefficient	17% improved **I_DC_:** over BS	2019
Yang et al. [141]	T1 MRI	Cross–level fusion with context (inference) network for stroke lesion segmentation (chronic)	DLF	ATLAS **I_DC_:** 0.58	2019
Qi et al. [142]	MRI	X–Net (a nonlocal operation to capture long–ranged dependencies) or the chronic stroke lesion segmentation	DLF	ATLAS **I_DC_:** 0.48	2019
Liu et al. [143]	MRI	multi-kernel DCNN with pixel dropout	DLF	SPES **I_DC_:** 0.79	2019
Chin et al. [144]	MRI	Cascaded Networks (U-Net)		Train (Private Dataset) **I_DC_:** 0.44	2020
Liu et al. [148]	MRI	Attention–based DRANet.	DLF	(748 Images Sub-acute) **I_DC_:** 0.76 (Best)	2016
ZHANG et al. [149]	DWI	A triple–branch DSN architecture with a multi–plane fusion network	Customized Loss Function	ISLES 2015 SSIS **I_DC_:** 0.62	2020
Amin et al. [150]	MRI	Auto encoders [segmentation]		**I_DC_**: 0.96 (BRATS)	2020
Bui et al. [151]	MRI	3D Dense Net	modified DLF	MRBrainS18 **I_DC_** 0.87	2019
Joshi et al. [152]	DWI-MRI	Dilated and Transposed CNN	Binary cross entropy plus the dice loss	ISLES 2015–2017 (train 25000, validation 4000) **I_DC_**: 0.85 (validation) and **I_JACD_**: 0.78	2018
Gupta et al. [153]	MRI	Multi–Sequence Network architecture: Conv. Layers, Pooling Layers (2 × 2), Up sampling layers (2 × 2), Dropout Layers,	Binary Cross–entropy	ISLES 2015 Core Esti **I_DC_:** 0.68 Penumbra Esti. **I_DC_:** 0.82	2019
Kumar et al. [154]	MRI	Classifier–Segmenter network (modified UNet for segmentation)	multi–scale loss function (customized)	ISLES 2017–SPES dataset **I_DC_:** 0.83	2020
Satish et al. [155]	DWI, PWI	Adversarial Architecture: Encoder–decoder as segmentor. Discriminators: CNN	cross–entropy	ISLES 2015 **I_DC_:** 0.82	2020

**DLF**, dice loss function; **I_ACC_**, accuracy; **I_ICC_**, intra class correlation coefficient; **I_PREC_**, ischemic precision; **I_FS_**, ischemic FScore; **I_SPEC_**, ischemic specificity; **I_SENS_**, ischemic sensitivity.

**Table 8:** Summary of various methods applied for stroke detection (hemorrhagic).

Modality	Articles	Technique	Loss Function	Outcome	Year
CT	Phong et al. [156]	LeNet, GoogLeNet, and Inception–ResNet Private Dataset of 1700 records		F1 Score 0.997 (LeNet)	2017
CT	Majumdar et al. [157]	9 (3 × 3) convolutional blocks, (2 × 2) max–pooling, BN and ReLU		81% **H_SENS_** per lesion 98% **H_SPEC_** per case	2018
NCCT	Patel et al. [160]	CNN with two distinct pathways integrating contextual information	categorical cross entropy	**H_DC_:** 0.91	2019
CT	Cho et al. [161]	FCN–8s		**H_ACC_:** 98.28%	2019
CT	Patel et al. [162]	CNN and RNN	Binary Cross Entropy	**H_ACC_:** 0.87	2019
NCCT	Barros et al. [163]	CNN		**H_DC_:** 0.63 ± 0.16	2020
NCCT	Lee et al. [164]	CNN		**H_AUC:_** 0.903	2020
CT	Xu et al. [165]	Masked RNN and ML		Model[Resnet50+ MLP+MobileNET] **I_ACC_**: EM 99.89 [Best]	2020
CT	Li et al. [166]	Pre–trained Dilated UNet		**H_DC_:** 0.8033	2021
NCCT	Arab et al. [167]	U–Net with deep supervision. Encoder: Residual block Decoder: Convl layers	Dice similarity coefficients	**H_DC_:** 0.84 ± 0.06	2020
CT	Grewal et al. [168]	Recurrent Attention DenseNet, bidirectional LSTM layer		**H_ACC_:** 0.8182	2018
NCCT	Burduja et al. [169]	CNN & LSTM	Binary cross–entropy	**H_LL_:** 0.04989	2020

**ICC**, infraclass correlation coefficients; **H_DSC_**, dice similarity coefficient; **ASSD**, average symmetric surface distance; **H_LL_**, log loss; **H_SENS_**, Sensitivity, **H_SPEC_**: Specificity.

**Table 9:** Summary of different methods applied for prognosis of strokes.

Articles	Modality	Technique	Prediction	Year
Rebouças et al. [51]	CT	Feature extraction based on density patterns (radiological) and classification of strokes through kNN, SVM, MLP, OPF and Bayesian classifiers	Identify & classify the occurrence of strokes (extent and severity).	2017
Robben et al. [84]	CTP	Modifed DeepMedic	Final infarct volume	2019
Bentley et al. [97]	CT	SVM with an **H_AUC_**: 0.744	Predict symptomatic intracranial hemorrhage	2014
Stier et al. [117]	Tmax MRI	**CNN** with 2 Conv layers, 2 6x6 pooling layers, trained with 100 epochs for Binary prediction	Tissue Fate Features in AIS	2016
Choi et al. [119]	MRI	Lesion outcome prediction—3D Res U–Net—CNN Clinical outcome prediction–CNN–Log Regression	Automated prognosis for post–treatment ischemic stroke	2016
Chen et al. [121]	CT	CNN	Early stroke detection (ischemic) system with CNN	2017
Lucas et al. [122]	CT	3D U–net appended with Convolutional auto–encoder		2018
Lucas et al. [125]	CT	3D UNets	Predict Ischemic Stroke Growth	2018
Bento et al. [126]		SVM **I_ACC_**: 97.5%	Early identification of Carotidartery Atherosclerosis	2019
Song et al. [127]		GAN **I_DC_**: 0.624	Prediction of perfusion parameters	2019
Giacalone et al. [124]		SVM **I_PRES_**: 95%	Final lesion prediction	2018
Arbabshirani et al. [158]	CT	DCNN **H_AUC_:** 0.846	Detecting of ICH based on clinical database of brain CT images	
